# Acute stress selectively impairs learning to act

**DOI:** 10.1038/srep29816

**Published:** 2016-07-20

**Authors:** Archy O. de Berker, Margot Tirole, Robb B. Rutledge, Gemma F. Cross, Raymond J. Dolan, Sven Bestmann

**Affiliations:** 1 Sobell Department of Motor Neuroscience and Movement Disorders, Institute of Neurology, University College London, WC1N 3BG UK; 2 Wellcome Trust Centre for Neuroimaging, University College London, WC1N 3BG UK; 3 Max Planck University College London Centre for Computational Psychiatry and Ageing Research, WC1B 5EH UK; 4 Clinical Biochemistry, King’s College Hospital, Denmark Hill, SE5 9RS UK

## Abstract

Stress interferes with instrumental learning. However, choice is also influenced by non-instrumental factors, most strikingly by biases arising from Pavlovian associations that facilitate action in pursuit of rewards and inaction in the face of punishment. Whether stress impacts on instrumental learning via these Pavlovian associations is unknown. Here, in a task where valence (reward or punishment) and action (go or no-go) were orthogonalised, we asked whether the impact of stress on learning was action or valence specific. We exposed 60 human participants either to stress (socially-evaluated cold pressor test) or a control condition (room temperature water). We contrasted two hypotheses: that stress would lead to a non-selective increase in the expression of Pavlovian biases; or that stress, as an aversive state, might specifically impact action production due to the Pavlovian linkage between inaction and aversive states. We found support for the second of these hypotheses. Stress specifically impaired learning to produce an action, irrespective of the valence of the outcome, an effect consistent with a Pavlovian linkage between punishment and inaction. This deficit in action-learning was also reflected in pupillary responses; stressed individuals showed attenuated pupillary responses to action, hinting at a noradrenergic contribution to impaired action-learning under stress.

Stress is linked to a cascade of changes in central and peripheral physiology, including a rapid rise in catecholamines and a sustained increase in glucocorticoids^1^. Stress also induces a shift in instrumental learning from flexible (model-based) systems towards an inflexible, and experience-dependent (model-free), form of control^2^,^3^,^4^,^5^,^6^. The degree of shift depends upon working memory capacity^5^, consistent with the idea that an impaired allocation of cognitive resources is a key mediating mechanism^2^,^3^,^4^,^5^,^6^,^7^.

However, recent work has also highlighted the importance of non-instrumental influences upon choice. Pavlovian responses resulting from the prediction of reward and punishment interfere with the learning of instrumental contingencies^8^,^9^,^10^,^11^. For example, this innate coupling between reward and approach is of such potency that chicks are unable to learn to walk away from a food source in order to harvest it^12^. An opposite linkage, between punishment and inaction, is evident in the freezing behaviour (inaction) seen across a wide variety of species^13^.

Pavlovian approach behaviour is also thought to underlie aspects of cue-triggered relapse in addiction^14^, an influence potentiated by stress^15^ via putative corticosteroid-dopaminergic interactions^16^. The observation that stress also engenders fast, context-inflexible, forms of control^2^,^3^,^4^,^5^,^6^, raises the possibility that its impact on instrumental behaviour is mediated via Pavlovian biases. Limited evidence in rodents^17^ suggests that stress transiently attenuates the impact of reward prediction upon vigour (Pavlovian-instrumental transfer, PIT), although other studies failed to observe an effect^18^. However, stress itself constitutes a sustained aversive state. Given the linkage between negative valence and inaction^11^, this suggests an alternative hypothesis, namely that stress impacts on instrumental learning via the Pavlovian coupling of punishment and inaction. Such a coupling would explain the augmented inhibition of pre-potent actions observed under threat of shock^19^, and predicts that sustained activation of punishment-related tendencies (such as active avoidance, or inaction^11^) might interfere with the learning of instrumental responses which required different behavioural outputs (such as approach).

To distinguish between these two competing hypotheses we compared a Stressed group who underwent the socially-evaluated Cold Pressor Test (CPT), and a Control group who submerged their hands in room-temperature water^5^,^6^,^7^ (Fig. 1A). We measured subjective stress and salivary cortisol at multiple time-points, allowing us to track stress levels throughout the experiment. Additionally, we recorded pupil diameter throughout, as an indirect assay of noradrenergic activity^20^,^21^, which has a central role both in action initiation^22^ and the co-ordination of stress responses^23^.

On each trial, participants saw a cue, and had to produce or withhold an action (Go/NoGo) so as to gain a reward (Win) or avoid a punishment (Avoid Loss) (Fig. 1B). The design was factorial, giving four conditions: Go to Win, Go to Avoid Losing, NoGo to Win, NoGo to Avoid Losing. The contingencies were probabilistic, such that the correct action led to the better outcome in 80% of cases. This task can be understood as comprising two Pavlovian-congruent conditions, in which the instrumental requirements match the valence-evoked Pavlovian resposnes (Go to Win and NoGo to Avoid Loss), and two Pavlovian-incongruent conditions, where the instrumental and Pavlovian responses are in conflict (NoGo to Win and Go to Avoid Losing). This task therefore provides a well-validated assay of Pavlovian biases, assessed by comparison of performance in the Pavlovian-congruent conditions, on which people perform well, and Pavlovian-incongruent conditions, on which they perform poorly^8^,^9^. This enabled us to ask whether stress affected Pavlovian biases over learning, or whether its effects were better described by a specific bias towards inaction, as suggested by an inhibition account of stress effects upon behavior.

## Results

### Stress induction

Contrasting the stressful CPT condition with the Control condition revealed a profound effect of our manipulation upon subjective stress, autonomic, and glucocorticoid activity (Fig. 2). Subjective stress ratings increased in the stressed group following immersion, and remained elevated above baseline throughout the task (Fig. 2A). The manipulation did not elicit a change in subjective stress in the Control group, yielding an interaction between Group and Time (Repeated measures ANOVA, Effect of Time: F_5,58_ = 7.31, η^2^ = 0.037, p < 0.001; Effect of Group: F_1,58_ = 2.01, η^2^ = 0.021, p = 0.16; Interaction: F_5.58_ = 10.05, η^2^ = 0.051, p < 0.001). In both groups, stress at the end of the experiment was greater than at baseline (paired t-test, Stressed T_29_ = 3.89, d′ = 0.71, p < 0.001; Control T_29_ = 2.64, d′ = 0.48, p = 0.013).

Similarly, cortisol concentrations diverged between the two groups over time (Repeated measures ANOVA, Effect of Time: F_4,58_ = 4.97, η^2^ = 0.022, p < 0.001; Effect of Group: F_1,58_ = 2.44, η^2^ = 0.028, p = 0.12; Interaction F_4.58_ = 9.2, η^2^ = 0.040, p < 0.001) in the manner predicted from previous work^5^,^7^ (Fig. 2B). Importantly, cortisol concentrations in the stressed group were still higher than those in the Control group at the end of the Go/NoGo task, consistent with a persistence of stress throughout the task (two-sample t-test at T30 following task completion, T_58_ = 2.76, d′ = 0.72, p = 0.0067).

Pupil diameter reflected stress induction (Fig. 2C). Pupil diameter immediately preceding immersion was similar between groups with no difference at baseline (two-sample t-test, T_57_ = 0.87, p = 0.38). In the Control group, pupil diameter rapidly fell over the duration of immersion. However, the Stressed group showed a sustained elevation of pupil diameter, an effect also present in the post immersion period, producing a significant interaction between group and time (Repeated measures ANOVA, Effect of Time: F_2,57_ = 55.61, η^2^ = 0.017, p < 0.001; Effect of Group: F_1,57_ = 0.15, η^2^ = 0.0025, p = 0.70; Interaction: F_2,57_ = 32.78, η^2^ = 0.10, p < 0.001). Treatment-induced changes in autonomic nervous activity were also evident in our measurements of blood pressure before and after stress induction (Table 1). Systolic and diastolic blood pressure after treatment were both higher in the Stressed group relative to Control (Systolic: Repeated measures ANOVA, Effect of Time: F_1,56_ = 2.57, η^2^ = 0.005, p = 0.11; Effect of Group: F_1,56_ = 1.30, η^2^ = 0.020, p = 0.26; Interaction: F_1,56_ = 6.6, η^2^ = 0.013, p = 0.013; Diastolic: Repeated measures ANOVA, Effect of Time: F_1,56_ = 0.13, η^2^ = 0.0004, p = 0.72; Effect of Group: F_1,56_ = 0.08, η^2^ = 0.0011, p = 0.78; Interaction F_1,56_ = 6.28, η^2^ = 0.022, p = 0.015). Heart rate showed a trend towards a difference between groups and an interaction with time (Repeated measures ANOVA, Effect of Time: F_1,56_ = 9.46, η^2^ = 0.0082, p = 0.0032; Effect of Group: F_1,56_ = 3.60, η^2^ = 0.57 p = 0.063; Interaction F_1,56_ = 3.45, η^2^ = 0.003, p = 0.069).

Finally, we examined the relationship between our three primary stress measures (Fig. 2D). Partial correlations indicated that manipulation-elicited change subjective stress was related to both AUC cortisol (r_Subjective-Cortisol_ = 0.39, p_Subjective-Cortisol_ = 0.0030) and change in pupil diameter (r_Subjective-Pupil_ = 0.41, p_Subjective-Pupil_ = 0.0019). However, pupillary and cortisol responses were not themselves related (r = 0.056, p = 0.68). This suggests that the subjective response to stressors may capture elements of the immediate catecholaminergic response reflected in pupil diameter, whilst also predicting the extent of delayed glucocorticoid release. We note that this is a relatively rare example of concordance between multiple stress measures^24^.

### Stress selectively impairs performance in learning to Go

Across all subjects, task performance was comparable to that seen in previous experiments^9^,^25^. Subjects performed best in the Go to Win condition (average correct = 87.6%), with poorer performance on Go to Avoid Losing (77.0%) and NoGo to Avoid Losing (77.83%), with the worst performance observed in the NoGo to Win condition (58.3%), replicating previous studies^11^. An ANOVA confirmed the action by valence interaction predicted from previous studies, along with main effects of action (participants found it easier to learn to Go than NoGo) and valence (performance was better in the Avoid Losing conditions) (Repeated Measures ANOVA, Effect of Action: F_1,59_ = 238.27, η^2^ = 0.32, p < 0.001; Effect of Valence: F_1,59_ = 83.82, η^2^ = 0.028, p < 0.001; Interaction F_1,59_ = 35.62, η^2^ = 0.28, p < 0.001). We gathered a questionnaire measure of impulsivity (Urgency, Premeditation, Perseverance, Sensation seeking; UPPS^26^), which we hypothesized might relate to behavior in the task due to its association with dopaminergic tone^27^. This was not the case; impulsivity was not related to the number of Go responses emitted during the experiment (r = 0.19, p = 0.15) or to the Pavlovian performance bias in the task (r = −0.064, p = 0.62).

To visualize differences between groups and conditions, we plotted the average choices across subjects, smoothed with a 5 trial window (Fig. 3A). Our results demonstrate a selective impairment in Go learning in the stressed group (Fig. 3B), the magnitude of which grew over time (Fig. 3C). Baseline-correcting for differences in Go responding at the start of the experiment in order to isolate changes in responding with learning, we tested for group differences over 10 time bins of 6 trials each. Stress decreased performance in the Go conditions at every time point (p between 0.0216 and 0.038, FDR correction for multiple comparisons applied). In order to confirm that this effect was not due to performance differences at baseline, we examined the number of correct responses made during the second-half of the experiment. The stressed group again showed reduced performance in the Go conditions (two-sample T-test, T_58_ = 2.17, p = 0.034). Mindful of artefacts in group-averaging^28^, which can produce average curves suggesting incremental learning despite discrete jumps in performance at the individual level, we also performed non-continuous classification of subjects as learners and non-learners (see Methods). This allowed us to ask whether the percentage of participants successfully learning in each condition differed by group. This analysis also showed that stress impaired learning in Go to Win (Learners in stress group: 26, Learners in control group: 30, X^2^ = 4.2, p = 0.038) and Go to Avoid Losing (Learners in stress group: 25, Learners in control group: 30, X^2^ = 5.45, p = 0.020) but not in either of the NoGo conditions (NoGo to Win Learners in stress group: 19, Learners in control group: 18, p = 0.79; NoGo to Avoid losing Learners in stress group: 26, Learners in control group: 26 p = 1).

Our results suggest a specific deficit in Go-learning following stress, in accordance with the hypothesis that the aversive nature of stress leads to a bias towards inaction. Our results do not support the alternative hypothesis of a general increase in Pavlovian biases in the Go/NoGo task. Performance on the NoGo to Win condition did not differ between groups, and the decrement in the Go to Win condition is incompatible with an increase in bias, which should lead to improvement on the Pavlovian congruent conditions (Go to Win and NoGo to Avoid Losing). We further confirmed that the Pavlovian performance bias, which quantifies the difference between responding on congruent and incongruent trials (see Methods) did not differ between the two groups (p = 0.98, T_58_ = 0.02, d′ = 0.0063).

To bolster this conclusion, we fit a reinforcement learning model that allowed us to isolate the Pavlovian interaction between action and outcome valences on trial-by-trial learning (see Methods and^25^ for a description of the model). No parameters differed between groups (Learning rate T_58_ = 1.54, p = 0.13; Reward Sensitivity T_58_ = 0.38, p = 0.71; Punishment Sensitivity T_58_ = −0.99, p = 0.33; Action Bias T_58_ = −0.078, p = 0.93; Pavlovian Bias T_58_ = 1.55, p = 0.12; Noise T_58_ = 1.80, p = 0.078) (Fig. 4A,B). Following a recent study which found no effect of stress upon model-based learning but did observe a relationship with cortisol concentration changes^5^ we looked for a correlation between model parameters and cortisol change (quantified by Area Under Curve, AUC, equivalent to the integral of cortisol changes over time)^29^. No correlations between cortisol change and model parameters were evident (Fig. 4C) (Learning rate r = 0.20, p = 0.12; Reward Sensitivity r = 0.097, p = 0.46; Punishment Sensitivity r = −0.12, p = 0.37; Action Bias r = −0.18, p = 0.18; Pavlovian Bias r = 0.17, p = 0.20; Noise r = 0.18, p = 0.16). Our reinforcement learning model did, however, provide us with trial-by-trial estimates of surprise for each subject, which we used to examine the relationship between stress and pupil responses during the task.

### Stress alters the effect of action upon arousal

Evidence suggests that pupillary responses relate to both action and outcome processing^30^,^31^,^32^, echoing noradrenergic responses^22^. Since noradrenergic dynamics are profoundly altered by stress^23^, we asked whether stress might influence the representation of action or outcome at the level of pupillary response, consistent with deficits in Go learning in the stressed group.

Our task elicited reliable fluctuations in pupil diameter, which depended both upon action and valence (Fig. 5A). Action produced a robust increase in pupil diameter, and this was further enhanced by an anticipation of loss. In light of our finding that Go learning is specifically impaired in stress subjects, we isolated an effect of action using multiple regression models aligned to target presentation (see Methods). Since stressed subjects performed worse in the two Go conditions relative to controls, they experienced a greater number of suboptimal outcomes and, on average, larger prediction errors (due to surprising outcomes). To account for this we included outcome valence and surprise (absolute prediction error as provided by our reinforcement learning model) on each trial in the regression, along with the action taken (Go or NoGo).

Action exerted a large effect upon pupil diameter both preceding and following target presentation in both groups, as captured by large positive regression coefficients (β) (average β 1–2 s following target, T_59_ = 8.58, p < 0.001) (Fig. 5B). This captures the difference between Go and NoGo conditions depicted in Fig. 4A. However, the magnitude of this action-induced dilatation differed between groups (Fig. 5F). Taking the maximum β for each subject, Stressed subjects displayed a smaller increase in pupil diameter in Go vs. NoGo conditions, as captured by a smaller β relative to Control subjects (T_58_ = −2.32, p = 0.024). This aligns with our behavioural observation that stress induces an action-dependent deficit in learning.

Our behavioural data suggests that stress-induced impairments are valence independent, and that Pavlovian biases within the task are unaffected by stress. To specifically examine valence and Pavlovian effects, we used an outcome-aligned regression model (see Methods), including terms for action, valence, their interaction (capturing Pavlovian biases), as well as the surprise induced by an outcome (extracted from reinforcement learning models for each participant). All three exerted systematic influences upon pupil diameter (Fig. 5C–E). Valence (whether an outcome was positive or negative) induced both anticipatory (average β 0–1s preceding outcome, T_59_ = −3.48, p < 0.001) and post-outcome (average β 1–2 s after outcome, T_59_ = −7.09, p < 0.001) effects (Fig. 5C). Since valence was coded as 1, 0, or −1, corresponding to Win, No Change, or Loss respectively, the negative value of coefficients implies a larger pupil in anticipation of, and following, monetary losses compared to gains of equivalent magnitude. The size of this effect did not differ between groups (T_58_ = −0.83, p = 0.41), as predicted from our behavioural finding of an action-specific, valence-independent effect of stress. The interaction of action and valence was significant post-outcome (average β 1–2 s after outcome, T_59_ = −2.62, p = 0.011) echoing a well-established interaction between action and valence in behaviour^9^,^11^,^33^. This also validates reinforcement learning models which localise this interaction to a post-outcome updating step (see Methods and^11^,^15^) (Fig. 5D). In line with our behavioural and modelling results, which implied that stress did not affect the expression of Pavlovian biases within the task, there was no difference in the magnitude of this interaction between groups (T_58_ = 0.25, p = 0.81) (Fig. 5F).

Several reports^30^,^31^,^32^ have highlighted a correlation between pupil diameter and trial-by-trial estimates of surprise inferred from computational models. We quantified surprise as the absolute magnitude of the prediction errors^30^ used to update beliefs in Q-learning models, as employed here^11^,^34^. We replicated previous findings that surprise exerts a positive influence upon pupil diameter post-outcome (average β 1–2 s after outcome, T_59_ = 2.72, p = 0.0086)^30^,^31^,^32^ (Fig. 5E). This effect did not differ between groups (T_58_ = −1.08, p = 0.29) suggesting that the feedback signals used in error-driven learning were not altered by stress (Fig. 5F).

## Discussion

We tested two hypotheses regarding the impact of stress on learning. First, stress might induce a greater dependence upon Pavlovian biases, in line with the idea of a stress-induced general shift from computationally demanding flexible systems towards more automatic forms of control^2^,^3^,^4^,^5^,^6^. Second, an alternative account suggests that stress facilitates punishment-related behaviours, as indexed by a shift towards inaction^19^. We found evidence supporting this second hypothesis; stressed participants were impaired in responding to both Go cues, and showing no deficit for both NoGo cues (Fig. 3), a conclusion supported by reinforcement learning models (Fig. 4). This impairment in learning to act is reflected by pupillary responses in stressed subjects, who showed attenuated pupillary responses to action whilst displaying no differences in the response to outcome valence or surprise (Fig. 5).

We note, however, that we do not observe an improvement in NoGo learning in the Stressed group, arguing against a global shift towards inaction. Stress thus appears to induce a specific deficit in action learning, whilst leaving intact the ability to learn to withhold an action. The specificity of our findings enables us to rule out several alternative explanations. Firstly, average performance on the NoGo conditions (67.6%) was lower than in the Go conditions (82.7%). Stressed subjects were therefore impaired on the *easier* of the two actions, ruling out the possibility that stressed subjects exhibit a difficulty-dependent deficit. Secondly, performance under stress in NoGo conditions was indistinguishable from the control condition, precluding a general performance deficit and underlining that stressed subjects were no more likely to correctly withhold a Go response in NoGo conditions. Thus, stressed subjects were not merely more likely to withhold actions; they were equally likely to produce them (incorrectly) in the NoGo conditions. Thus, the deficit we describe is learning-specific and unlikely to reflect an impaired production of action by stress, but instead reflects an impairment in action learning from reinforcement. We note here that motor *excitability* is enhanced by cortisol administration^35^, but motor *plasticity* is inhibited^36^. These observations underline a potentially pertinent difference between simple action production and action learning under stress.

This distinction is also important for interpreting the results of the reinforcement learning model (Fig. 4). This model quantifies the Pavlovian bias in learning, crystallized in a parameter estimate for each participant. This parameter is affected by dopaminergic maniplations^25^ and related to midline-theta activity in EEG experiments^37^. We found that this parameter was unaffected by stress, bolstering our rejection of the hypothesis that stress amplifies within-task Pavlovian biases. The model failed to capture the effect we observed, namely a selective deficit in action-learning. This is unsurprising; the model explains interindividual differences in the production of action in terms of an action bias parameter, but it cannot capture specific deficits in action *learning*. Our results suggest that expansion of the model to capture action-specific learning deficits would be fruitful.

The influence of action upon pupil diameter was attenuated in stressed subjects (Fig. 5B,F). Pupil diameter is frequently described as an index of noradrenergic activity^20^,^30^. This link is reinforced by observations in a non-human primate study in which specific correlations were observed between pupil diameter and activity of single neurons in the noradrenergic locus coeruleus, but not the dopaminergic substantia nigra pars compacta^21^,^22^. Numerous reports of interactions between glucocorticoid and noradrenergic systems^11^,^38^,^39^,^40^,^41^ suggest plausible substrates for altered pupillary responses under stress. For example, a stress-induced increase in glucocorticoids might affect noradrenergic activity in manner that specifically impacted learning following actions. Another possibility is that altered pupillary responses in the Go conditions is a *consequence* of the inability to learn. However, by including surprise as a regressor in our model of pupil diameter we show this did not account for group differences, despite an overall positive effect of surprise upon pupil diameter post-outcome (Fig. 5E), in line with previous reports^30^,^31^,^32^.

We observed an interaction between action and valence in pupillary responses (Fig. 5D). Although fMRI studies have highlighted an interaction between action and valence in basal ganglia responses^9^, we believe ours is the first physiological data with the necessary temporal precision to offer insight into the belief-updating process hypothesized to underlie this task. Computational models of learning in the Go/NoGo task place this interaction at the point at which action-weights are updated, precisely in the time window in which we observe such an interaction (for a summary of such models, see^11^ Box 1 and Methods). Our physiological evidence for action-valence interactions following outcome thereby validates modified reinforcement learning models which incorporate Pavlovian influences in a post-outcome updating term.

We used a common laboratory stressor, the CPT, to manipulate stress levels^5^,^6^,^7^. This produced both an immediate increase in subjective stress and physiological arousal (Fig. 2A,C), and a delayed, sustained increase in glucocorticoid concentrations (Fig. 2B). Subjective stress returned swiftly to baseline following the manipulation, mirroring recent findings that subjective states such as stress reflect recent events with a relatively fast decay constant^42^,^43^. We do find, however, that subjective stress response to the manipulation predicts the magnitude of the sustained glucocorticoid increase that follows it (Fig. 2D), suggesting a degree of concordance between the severity of the subjective experience of stress and its physiological sequelae, which are presumably responsible for the protracted behavioural effects we observe here.

A recent study used a reward-based paradigm in which participants performed certain actions to obtain stimulus-paired confectionary rewards from a ‘vending machine’^44^. This allowed them to assess both the enhancement of a certain action given the presence of the reward-cue associated with that action (specific Pavlovian-to-Instrumental Transfer, PIT), and the general increase in responding accompanying the presence of a reward-associated cue (general PIT). They observed effects of chronic stress (assessed with a questionnaire measure) upon general transfer; highly stressed participants did not respond more in the presence of a reward-related cue. Although the similarities between that study and this one should not be overstated – there are considerable differences between the acute stress manipulation employed here and the and assessment of chronic stress levels used there- this blunting of reward-related action production may relate to the deficit in action-learning we observe. In both cases, stress is associated with a reduced tendency to produce actions associated with positive reinforcement.

The reported effects of stress upon learning are famously variable^45^. One potential explanation for this heterogeneity is that superficially similar behaviours are supported by distinct neural computations, which exhibit contrasting responses to stress. Recent work using choice between pairs of stimuli has suggested a shift towards the use of rewarding vs. punishing feedback during learning^46^,^47^. By contrast, we observe a valence-independent deficit in a task where participants choose to produce or withhold a response to a single stimulus. Such subtle differences in choice reference frame can have dramatic impact upon the neural circuits recruited during choice^48^. Specific stressors may also differ in the effects that they produce. For example, the CPT requires participants to suppress an action (the withdrawal of the hand from the ice bucket), which could conceivably prime the suppression of action-learning we observe.

Exposure to chronic uncontrollable stress induces an inability to learn to avoid future punishment, an effect described as ‘learned helplessness’^49^. However, this effect also holds when the agent is required to learn NoGo responses to avoid punishment, suggesting that it is distinct from the deficit we observe here^50^. Learned helplessness is perhaps best described as a consequence of generalisation from one episode to another^51^. It is not clear how generalisation from events in the Cold Pressor Test to learning in our Go/NoGo learning task might underpin the effect we describe here, though we consider it a possibility.

What might be the functional impact of stress inhibiting learning to act? One possible explanation is that stress is typically associated with periods of high metabolic demand and uncertainty^52^. In situations where you are unsure what to do, doing nothing has the immediate advantage of being metabolically inexpensive. In the aftermath of an acute stressor such as the one we deploy here, biasing learning away from energetically costly activity may be a useful strategy for conserving resources.

In many professions, such as military, financial, or emergency medical services, sporadic surges in cortisol levels are the norm^53^,^54^,^55^. Our data suggest that whether an option is selected by action or inaction might have an important role in learning from decisions made under stress. One prediction is that stressed people should manifest a greater reliance upon default options, a consequence of an impaired ability to learn from action relative to inaction. Such biases could theoretically be prevented by randomising action-outcome associations. For example, the performance of a stressed stock-trader might benefit from sometimes having selling a stock as the default option and keeping the stock requiring action.

## Materials and Methods

### Participants

We recruited 64 participants (32 males) through UCL’s Institute of Cognitive Neuroscience (ICN) database. Participants were screened for medical conditions, previous CPT exposure, and previous participation in any experiments involving the Go/NoGo task. 4 participants were excluded (distractibility during the experiment or misunderstanding of the task revealed upon debrief), leaving 60 participants (30 CPT, 30 Control, 15 males in each group).

All participants signed an informed consent form. All collected data was treated as strictly confidential and handled in accordance with the provisions of the Data Protection Act 1998. Medical supervision was present throughout. The protocol was approved by the UCL Research Ethics Committee (Ethics 4377/001). All experiments were conducted in accordance with approved guidelines.

### Procedure

**Figure 1:**
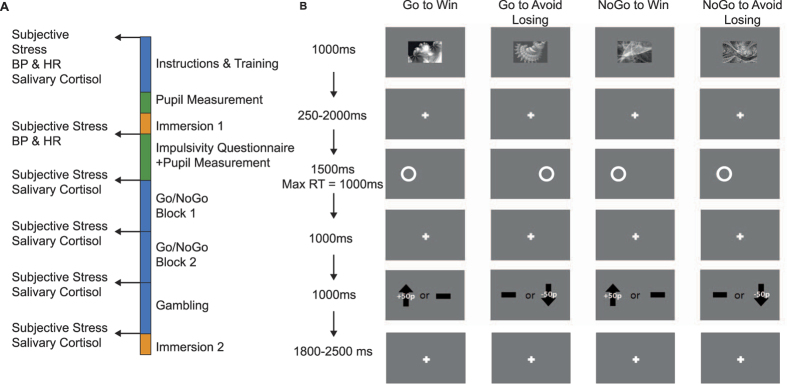
Experimental design. (**A**) Timecourse of the experiment. After instruction and training, subjects were asked to immerse their arms for three minutes in 0–1 °C (Stressed group) or 24–27 °C (Control group) water. Blood Pressure (BP) and Heart Rate (HR) were assessed before and after immersion. After a waiting period of ten minutes, they then completed the Go/NoGo task followed by a separate gambling task. At the end of the experiment, subjects underwent an expected second immersion lasting 30 seconds. Subjective stress and salivary cortisol were assayed regularly throughout the task. (**B**) The Go/NoGo task. On each trial, one of four cues was presented. Each cue was associated with an initially unknown correct action (Go or NoGo) and an outcome (Win money or Avoid Losing money). Subjects learned to select actions based upon the outcomes. Following the cue, a target was presented on the left or right side of the screen and subjects chose whether to press a button corresponding to the side of target presentation. For the cues associated with winning money, the correct choice was rewarded with an increase in earnings on 80% of trials. For cues associated with losing money, the incorrect choice was punished with a decrease in earnings on 80% of trials.

The experimental procedure is summarised in Fig. 1A. Participants read an instruction sheet describing the structure of the experiment, which specified whether they were in the CPT or Control group, and gave their informed consent. Participants then underwent basic computerized training in the Go/NoGo task and a separate gambling task (results not discussed here). They then provided a saliva sample and had their blood pressure taken, before a measurement of baseline pupil diameter. Participants then submerged an arm in ice-cold (Stressed condition) or room-temperature water (Control condition) [details below]. Following withdrawal of the hand from water, participants completed a questionnaire (Urgency, Premeditation, Perseverance, Sensation seeking; UPPS^26^), and then maintained fixation until 10 minutes had elapsed since the end of submersion. This period was chosen to accommodate the timecourse of glucocorticoid release, such that cortisol concentrations would be elevated when they began the Go/NoGo task^56^. Participants then performed the Go/NoGo task, with a self-paced break halfway through. They then performed a gambling task, the results of which will be presented elsewhere, before performing a final, brief submersion of 30 seconds.

### Stress manipulation & measures

We used the widely adopted Cold Pressor Test (CPT) to induce stress^5^,^6^,^7^,^57^,^58^. Water temperature was 0–1 °C in the Stressed condition, and 24–27 °C in the Control condition. Participants were asked to keep their arm submerged for 3 minutes. Participants in the Stressed condition were monitored by an additional experimenter, who entered the room specifically to observe this phase of the experiment, adding a social-evaluation component to the stressor^7^. All control subjects kept their arms submerged for the full 3 minutes, whilst several stressed participants were unable to remain submerged for the entire period (mean duration = 144 seconds, range 39–180 seconds). Subjects were informed at this point that they would be completing a second submersion at the end of the experiment, a manipulation designed to sustain negative affect throughout the task.

We measured stress responses in three ways, designed to capture the subjective response (assessed with visual analogue scale ratings) and physiological response to stress respectively. The latter can be fractionated into a rapid, catecholaminergic component (indexed by pupil diameter) and a slower, glucocorticoid release (indexed by salivary cortisol samples). We refer to the timings of cortisol and subjective stress assays with reference to the end of submersion and the approximate times for subsequent assays: T0, T10, T20, T30, T45. Cortisol was not measured at T0, as the sluggish dynamics of the HPA axis preclude an immediate glucocorticoid response to stress^59^. Pupil diameter was measured throughout, though measurements could not be obtained at hand immersion and withdrawal due to excessive movement.

Subjective stress was assayed using experiential sampling^42^,^43^,^60^, which involved each subject moving a cursor along a line to answer the question ‘How stressed do you feel right now?’, anchored by ‘Not stressed’ and ‘Very stressed’ at each extreme. Ratings for subjective stress from three participants were lost due to technical error.

Pupil diameter was measured using an EyeLink 1000 system, sampling at 250 Hz. The experiment took place in a darkened room, with a computer screen shielded on each side to minimise reflections. Participants were asked to maintain fixation wherever possible. All stimuli in the experiment were luminance matched within stimulus-type, although it was not possible to match luminance between stimuli. Data was subsequently exported to ASCII and imported to Matlab for analysis, where automatically-identified blink events were removed and replaced via linear interpolation of samples 140 ms either side of the blink. Data were then low-pass filtered (2^nd^ order Butterworth Filter, 4 Hz) and z-scored before analysis^31^,^32^,^61^.

**Figure 2:**
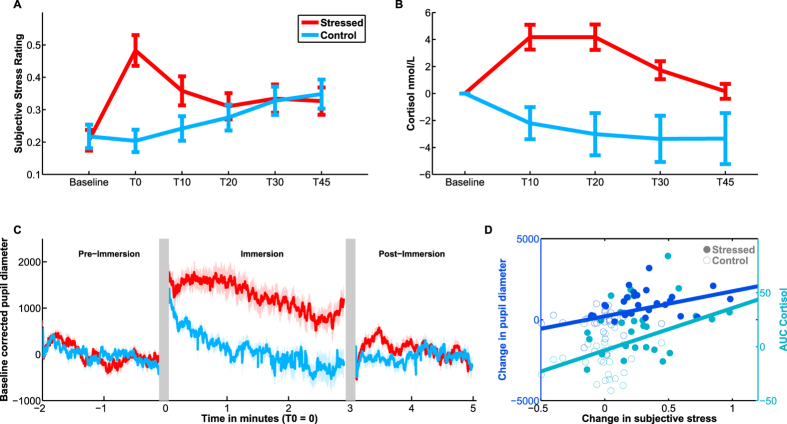
Confirmation of stress induction. (**A**) Subjective stress was assessed using a visual analogue scale. Immediately before timepoint T0, subjects immersed their hands in either very cold (0–1 °C, Stressed group) or room temperature water (25–28 °C, Control group). We observed an interaction between group and time, with an increase in subjective stress induced by the Cold Pressor Test relative to the control condition. (**B**) Salivary cortisol samples were taken at 10–15 minute intervals following immersion. We observed a robust and sustained increase in baseline-corrected salivary cortisol in the Stressed relative to the Control group, which persisted until the end of the experiment. Data baseline corrected for display. (**C**) We measured pupil diameter in a baseline period prior to T0, during immersion, and post immersion (see Fig. 1A). Grey rectangles: signal loss during insertion and withdrawal of the hand from water. Data baseline corrected for display. (**D**) Increase in subjective stress during immersion correlated both with the increase in pupil diameter during immersion and with the Area Under Curve (AUC) of cortisol increase (see Methods). Filled circles: stressed subjects; Open circles: control subjects, with dark blue corresponding to pupil measurements and cyan to cortisol measurements. Each participant contributes two data points (one dark blue, one cyan). All error bars are SEM across participants.

For analyses of stress effects upon pupil diameter (Fig. 2C) we measured pupil diameter in a baseline period prior to T0, during immersion, and post immersion. A single subject was excluded from this analysis due to complete data loss during immersion. Having removed periods of signal dropout, we then subtracted the average pupil diameter for each subject during the baseline period to correct for inter-individual variance in pupil size.

To examine the effect of task-events upon pupil diameter, we epoched the data in three ways: by Cue, by Target, and by Outcome. This was necessary as the timings of events within each trial varied according to imposed jitter and variable reaction time (Fig. 1B). In all cases, we accounted for drift in baseline pupil diameter over time by subtracting a baseline measurement of the average diameter for the first 200 ms following cue presentation, resulting in pupil diameter traces for all trials starting around zero.

We used multiple regression models to decompose the influence of different task events upon pupil diameter at multiple timepoints^30^. We used separate models in order to describe pupil diameter locked to Target presentation, and at Outcome. In both, we assessed the influence of action (Go/NoGo), outcome valence (Gain, No Change, Loss) and surprise (absolute prediction error from reinforcement learning models, see below) upon pupil responses. In the Outcome analysis, we also included an interaction term (Action*Valence). All predictors were z-scored before analysis to produce regression coefficients of comparable magnitude.

We decomposed each epoch into 40 time points (one every 200 ms), and performed a multiple regression analysis across trials at each time point using the robustfit function in Matlab. This allowed us to examine the influence of each of our predictors (action, valence, and surprise) upon pupil diameter at different times in the trial. Inference was based upon the distribution of regression coefficients (β) across subjects, where we test for differences from zero using single-sample t-tests. When testing for differences from zero (i.e. whether a predictor consistently affects pupil diameter) we take the average β coefficients for a period 1–2 s following outcome. For inference between individuals, we use the maximum absolute β across timepoints for each subject, allowing for interindividual variance in reaction time and pupillary dynamics.

For salivary samples, participants salivated through straws into 2 ml polypropylene tubes. Samples were frozen on the day of collection. Analysis was performed by Viapath at King’s College Hospital, using a competitive immunoassay. Briefly, cortisol in the sample competes with cortisol conjugated to horseradish peroxidase for binding sites on a microtitre plate. Unbound reagents are then washed away. Bound cortisol enzyme conjugate is measured by the reaction of the horseradish peroxidase enzyme to the substrate tetramethylbenzidine, producing a blue colour. A yellow colour is formed after stopping the reaction with an acidic solution. The concentration of cortisol in the sample is calculated as a function of the optical absorption at 450 nm; more absorption implies greater concentration of cortisol enzyme conjugate, and therefore lower concentration of cortisol in the sample (for further details see^62^). We quantified the experimental manipulation-evoked change in cortisol by calculating the Area Under Curve (AUC) with respect to baseline cortisol concentrations^29^. This standardised measure is a discrete equivalent of integrating the timecourse of cortisol concentrations.

### Go/NoGo task

The structure of the Go/NoGo task^9^,^25^,^33^,^37^ is depicted in Fig. 1B. Participants performed 240 trials. On each trial, 1 of 4 stimuli was displayed. Each stimuli denoted a different action/outcome contingency: Go to Win, NoGo to Win, Go to Avoid Losing, and NoGo to Avoid Losing. Following a variable interval, a target was presented on the left or right hand side of the screen, to which participants decided to respond (pressing the arrow button corresponding to the side of target presentation) or not. Following a brief fixation, the outcome was then presented. In the Win conditions, the correct choice was associated with a monetary gain on 80% of trials, and no change in earnings on 20%. In Avoid Losing trials, correct choice resulted in no change in earnings in 80% of trials, and a monetary loss in 20% of trials. Following incorrect choice, the outcomes were flipped, such that the worst outcome was received 80% of the time. Trials were separated with a variable fixation period of 1800–2500 ms.

**Figure 3:**
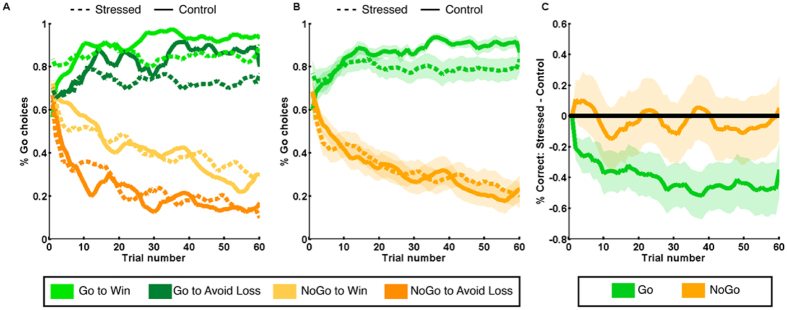
Stress impairs learning to act. (**A**) Percentage Go responses for each condition, for stressed subjects (dashed line) and controls (solid lines). (**B**) Grouping by Go and NoGo conditions (taking the mean across valences) reveals a deficit in Go learning in stressed subjects. (**C**) The difference in performance in Go conditions (averaged across valences) between groups, corrected for baseline differences in Go responding, grew throughout the experiment. All error bars are SEM across participants.

**Figure 4:**
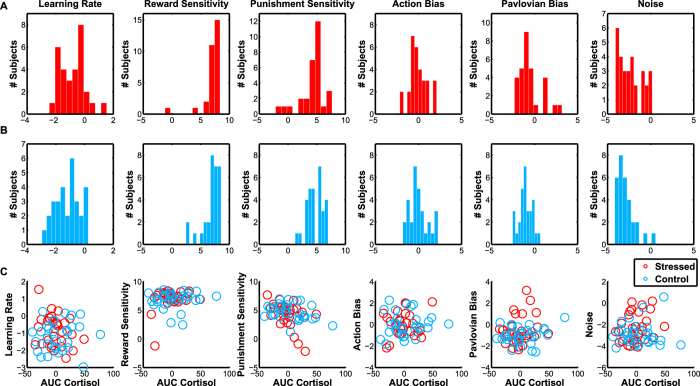
Stress does not affect the parameters of a Pavlovian learning model. (**A**) Distribution of parameters fit to responses from Stressed participants. (**B**) Distribution of parameters fit to responses from Control participants. (**C**) Relationship between AUC cortisol (see Methods) and model parameters. No correlations were significant. Each data point is a participant.

**Figure 5:**
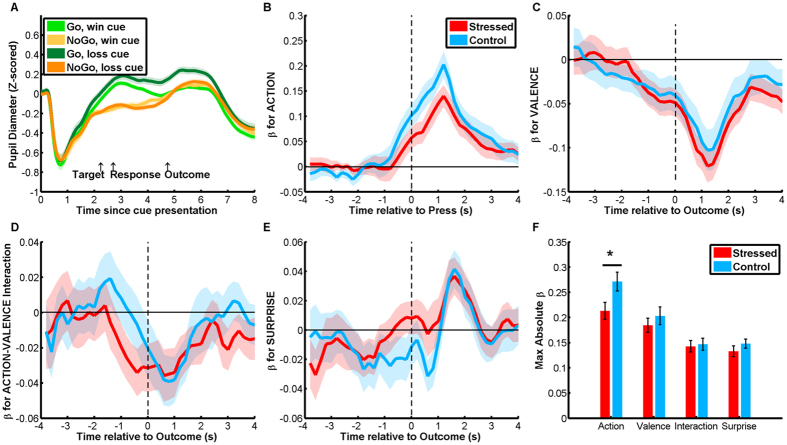
Stress alters pupillary responses to action. (**A**) Pupil diameter for Go and NoGo responses, separated by whether cue indicated the possibility for winning or losing money, and whether an action was produced. Action induces a robust increase in pupil diameter, amplified in trials involving the potential for monetary loss. (**B**) To isolate an effect of action upon pupil diameter, we constructed multiple regression models allowing us to account for between-group differences in outcome and surprise (see Methods). Aligned to target presentation, action exerts a large positive impact upon pupil diameter. On average, this effect was larger in the Control group (Fig. 4F). (C) We used an outcome-aligned regression model (see Methods) to examine the effect of outcome valence upon pupil diameter. Valence affected pupil diameter both in anticipation of and following outcome, with negative valence increasing pupil diameter. (**D**) The same outcome-aligned regression model as in C demonstrated a significant interaction of action and valence following outcome presentation. This effect was driven by a stronger differentiation of gain and loss following Go. (**E**) Surprise increases pupil diameter. Using the same outcome-aligned regression model as in C and D, surprise (absolute prediction error) induced an increase in pupil diameter. (**F**) Action-induced pupil dilatation is reduced by stress. To avoid multiple comparisons over time, we selected the maximum absolute β for each subject for each of our 4 regressors. We then asked whether any of the effects we observed differed by group. Only the β for action was affected by stress. Coefficients for valence, action-valence interaction and surprise did not differ between groups. All error bars are SEM across participants.

We analysed data from the task in four ways. Firstly, we examined average learning curves for each condition and in each group, following previous analyses^9^. In analyses where we present aggregates across conditions (e.g. Fig. 3B,C), we averaged across conditions (within subject) before computing between-subject averages. Where relevant we performed two-sample t-tests between groups, correcting for comparisons at multiple time-points using the Benjamini-Hochberg method^63^ to control the False Discovery Rate (FDR).

However, average learning curves can obscure more discrete differences between individuals who have learnt the task and those who have not^28^. To accommodate this, we also performed group comparisons based upon the number of participants who responded correctly in >50% of trials for each condition, testing for significance using chi-squared tests.

Thirdly, modified reinforcement learning models describe learning in the Go/NoGo task^11^ as a process of belief updating in each state on the basis of reward prediction errors, where the latter capture discrepancies between an action’s actual and expected consequences. To test for effects of stress upon the Pavlovian bias we fit a common variant of Q-learning^34^ in which action-values are biased by an interaction between action and state-value, amplifying weights on Go responses in the reward domain and suppressing them in the punishment domain (see^11^, Box 1 for a limpid description of this implementation, and below). We also use a representation of reward prediction error in this model to quantify and control for effects of surprise upon pupil diameter^30^,^31^.

Finally, the Pavlovian performance bias was calculated as described previously^37^ as the average of action invigoration in rewarded conditions ([Go in Go To Win + Go in NoGo to Win]/Total Go) and action suppression in punished conditions ([NoGo in Go to Avoid Losing + NoGo in NoGo to Avoid Losing]/Total NoGo). This provided a summary measure of how strongly action and valence interacted in choice.

### Structure of the Pavlovian bias model

Our model is based upon learning in 4 states – Go to Win, NoGo to Win, Go to Avoid Losing, No Go to Avoid Losing. These correspond to the 4 cues. For each state, there are two possible actions: Go and NoGo. We assume that on each trial the agent updates their beliefs about which action is better to take based upon Reward Prediction Errors (RPEs), which are calculated as the difference between outcomes and the agent’s expectation. Expectations are captured by Q-values (hence Q-learning). Q_t_(a,s) describes the Q-value of state *s* and action *a*, at time *t*. Q-values are updated via RPE’s, so following an action *a* in state *s:*


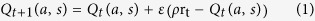


Where Q_t_ is the state value on trial t, ε is the learning rate, ρ is a weighting parameter which is multiplied by r_t_, the reward or punishment on that trial (taking a value of −1, 0 (no change) or 1). We allow ρ to vary, accommodating varying sensitivity to punishments and rewards (losing a given amount of money is typically more negative than acquiring the same amount of money). To reiterate, *a* and s denote the current action and the current state, respectively.

Action selection is based upon Q_t_(a,s); however, the model is ‘indirect’ in the sense that action weight are allowed to diverge from Q_t_(a,s); by the addition of two additional terms: an action bias, and a Pavlovian bias. This captures the intuition that action selection might be subject to a variety of biases that do not affect beliefs. Action weights are thus updated separately from Q_t_, following Q-updates, and contingent upon which action was selected:


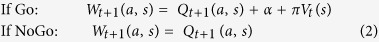


Where α is the action bias, π is the Pavlovian bias, and V_t_ is the current state value. The effect of this uncoupling between Q and W is to accommodate an action bias (α) (a general bias towards Go), and the Pavlovian interaction between action and valence, which emerges from the parameter π as follows. Go weights in rewarded states are amplified by the addition of the product of the positive parameter π and the positive state value 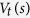. Conversely, negative state values decrease W_t+1_ for Go trials, through the addition of the product of the positive parameter π and the negative state value 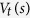. Note that unlike Q_t_, V_t_ is a function of the state alone, not contingent upon the currently selected action. V_t_ is updated through prediction errors in a manner analogous to Q_t_ but irrespective of action:


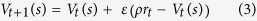


Where V_t_^(s)^ is the state value of state *s* at time *t*, ε is the learning rate, ρ is the reward/punishment sensitivity, and r_t_ is the outcome on that trial.

Action selection on each trial is based upon a comparison of action-weights within a squished-sigmoid, which allows the entry of irreducible noise (ξ) into action selection. This effectively prevents action selection becoming deterministic (all Go or all NoGo) even when the evidence in favour of one action over the other is comprehensively conclusive.


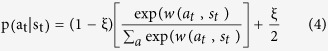


Where p(a_t_|s_t_) is the probability of selection an action *a* in a state *s* at time *t*, ξ is irreducible noise, and w(a_t_,s_t_) is the action weight for action *a* in state *s* at time *t*.

### Model fitting

The value of six parameters (ε, the learning rate; ρ_reward_, the reward weighting; ρ_punishment_, the punishment weighting; 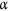, the action bias; π, the Pavlovian bias, and ξ, the irreducible noise in action selection) were fitted using Expectation Maximisation (EM) algorithms, as described in^64^. The reader is referred there for full details, although we sketch out the intuition below.

EM fitting occurs iteratively. The ultimate aim is to maximise the likelihood of *all* the data given a set of population parameters that are quantified by their mean (μ) and variance (σ). This approach differs markedly from a single-subject fitting algorithm, in which data from each subject would be described by a fitted parameter, resulting in a total of 360 free parameters. This renders the procedure highly vulnerable to overfitting. Conversely, we effectively fit a total of 12 parameters, a mean and a variance for each parameter. To achieve this, on each iteration of the model, the likelihood of a value for a given parameter is penalised by its improbability given the current distribution of parameters in the population. This effectively ‘pulls in’ extreme parameter values, which might fit a single subject’s data well, but are unlikely given the population distribution of parameter values. Note that we fit both groups as a single population, allowing us to compare parameters between groups using conventional statistics. All parameters were fit in an unbounded space (−∞  → +∞), and then transformed to a space of either 0 → +∞ (exponential transform, used for ρ, 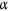, and π), or 0 → 1 (sigmoid transform, used for ε and ξ).

### Statistical analysis

Statistical tests are described at the point of use. We used parametric tests throughout.

## Additional Information

**How to cite this article**: de Berker, A. O. *et al*. Acute stress selectively impairs learning to act. *Sci. Rep*. **6**, 29816; doi: 10.1038/srep29816 (2016).

## Figures and Tables

**Table 1:** Cardiovascular stress measures.

	Systolic Blood Pressure (mmHg)		Diastolic Blood Pressure (mmHg)		Heart Rate (Beats Per Minute)	
	Mean Before (SEM)	Mean After (SEM)	Mean Before (SEM)	Mean After (SEM)	Mean Before (SEM)	Mean After (SEM)
Stress	116.17 (2.46)	116.87 (2.42)	68.72 (1.28)	71.0 (1.41)	71.52 (1.99)	68.27 (1.79)
Control	115.48 (2.28)	110.67 (2.25)	71.38 (1.53)	69.50 (1.21)	75.41 (1.97)	74.70 (1.85)
